# Design and fabrication of carbon fiber lattices using 3D weaving

**DOI:** 10.1038/s41598-023-40962-4

**Published:** 2023-09-10

**Authors:** Hayley McClintock, Zechen Xiong, Bruno Rergis, Hod Lipson

**Affiliations:** Fu Foundation School of Engineering and Applied Science, Columbia University, New York, NY 10027 USA

**Keywords:** Mechanical engineering, Structural materials

## Abstract

We present a method of designing and fabricating 3D carbon fiber lattices. The lattice design and fabrication is based on crocheting and sewing techniques, where carbon fiber tow is woven through two parallel carbon fiber grids and reinforced with vertical carbon fiber tubes. Compression testing is then performed on three different designs, and these results are compared to other similar lattice structures, finding that the lattices perform similarly to comparable lattices. Finite element analysis is also performed to validate the experimental findings, and provides some insight into the experimental results. The process presented here allows for more design flexibility than other current methods. For example, within a single lattice, different density weave patterns can be used to address specific load requirements. Though fabricated manually here, this process can also be automated for large scale production. With this design flexibility, simplified fabrication, and high strength, the lattices proposed here offer an advantage as compared to similar existing structures.

## Introduction

When designing structures for aerospace applications, it is critical to reduce mass while maintaining strength. To achieve this objective, lattice structures are often used as a way to engineer a low density material that maintains the desired mechanical properties. Lattice design and geometry has long been studied, and there are a number of geometries that yield high performance lattices, such as octet-truss^1^, tetrahedral^2,3^, pyramidal^4,5^, and 3D Kagome lattices^6,7^.

Traditionally, these lattices were made from metal, and were fabricated using traditional manufacturing methods such as investment casting^8^ and sheet folding^9–11^. Recently, carbon fiber has emerged as a superior alternative to metal for constructing lattices. With a higher strength to weight ratio, carbon fiber performs better than other previously used metals. Carbon fiber lattices can be fabricated using some of the same methods used for metallic lattices. Common techniques are usually cutting and folding^12,13^, snap fitting^5,14^, hot press molding^10,11,15^, or using mechanical fasteners^16,17^. One drawback with almost all of these fabrication methods is that they require manual assembly. This is time consuming and unwieldy, and is not practical for fabricating structures at larger scales.

One possible solution to this issue is to use additive manufacturing methods, such as 3D printing. By employing digital manufacturing processes, not only is fabrication time reduced, but errors introduced by manual assembly are eliminated as well. Moreover, the programmability of digital manufacturing processes allows for customization and optimization not afforded using bulk production processes.

Currently, there are a number of carbon fiber 3D printers commercially available. Many use chopped carbon fiber particles mixed with thermosets^18^, but some also use continuous carbon fibers^19,20^, though these fibers do not usually span across multiple layers. For example, Liu et al.^21^ demonstrated a free-hanging 3D printing technique that allows for the truss orientation to follow the axis of the slant of the struts. However, the 3D printing process can still be quite time consuming. Similarly, Eichenhofer et al.^22^ presented a pultrusion/extrusion method to additively manufacture lattices in a continuous manner with directed orientation of the fibers. Additionally, winding can also be used as a method to continuously produce lattice structures^23–25^. One drawback with these methods is that they do not allow for a robust connection between layers if a lattice was made with more than one unit cell in height.

In this research, we explore an alternative method to designing and fabricating carbon fiber lattices. Drawing inspiration from crocheting and sewing processes, the carbon fiber is woven through a diagonal carbon fiber grid held tightly in a jig. These taught threads are equivalent to the *warp* threads in a loom, except that they are interlaced into a lattice instead of being parallel. We then join lattice by weaving thread vertically, equivalent to the *weft* threads in a loom. The resulting 3D lattices then contain both diagonal and rectangular struts. Though previous work has demonstrated the effectiveness of woven lattices^26,27^, the method here offers the advantage of not needing a faceplate during fabrication. Without the restriction of the faceplate, more advanced designs can be created. For example, the warp planes do not need to be parallel to each other or even planar or uniform. Similarly, the weft threads can be non-uniform, optimizing strength to weight performance.

Here, after fabricating three different designs, the lattices are subjected to compression testing to determine the compressive strengths of the structures. The results are then compared to a finite element (FE) simulation to validate the experimental findings. Finally, the results of the compression testing are compared to similar structures.

## Methods

Carbon fiber tow is used as the main lattice material, as it is high strength and low weight, and is commercially available. The carbon fiber tow (HMT301-34/700 Tow Preg, Northern Composites) is pre-impregnated with resin, meaning that the tow can be directly cured without having to apply resin after shaping the material. The fiber volume ratio is taken to be 60% and the individual filaments that make up the tow are taken to be 5–8 µm^28^ The method described here is based on crocheting and sewing principles. First, an aluminum jig is fabricated that contains holes along each wall. Carbon fiber tow can then be woven through these holes to form a diagonal grid. Two layers of these grids are woven onto the jig to use as a substrate for the weaving process. Additionally, if desired, vertical, unidirectional, pultruded carbon fiber tubes can be added at each node of the grid by placing unidirectional carbon fiber tubes (McMaster-Carr) at the nodes and curing them in place with a small amount of epoxy. Here, two different tubes are studied; one with an outer diameter of 3 mm and an inner diameter of 2 mm, and another with an outer diameter of 8 mm and an inner diameter of 6 mm. Once the tubes are cured in place (or after the grid is woven if tubes are not being used), a latch hook is used to loop and knot carbon fiber tow around the grid. The latch hook is first lowered through both grid layers with the latch open. Then, the carbon fiber tow is wrapped around the hook, and the hook is pulled back up and the latch is closed.

Once pulled through, the latch hook is then moved above the next location and the process is repeated. Once the grid is in place, the lattices are cured at 230 °C for 20 minutes. After curing, the lattice is removed by cutting the connections to the jig. Both the weaving process and the removal process can be automated in future iterations of this project. A final step of dipping the entire structure in epoxy after the initial curing is also added to increase the strength of the lattice. An overview of this process is shown in Fig. 2.

## Results

**Figure 1:**
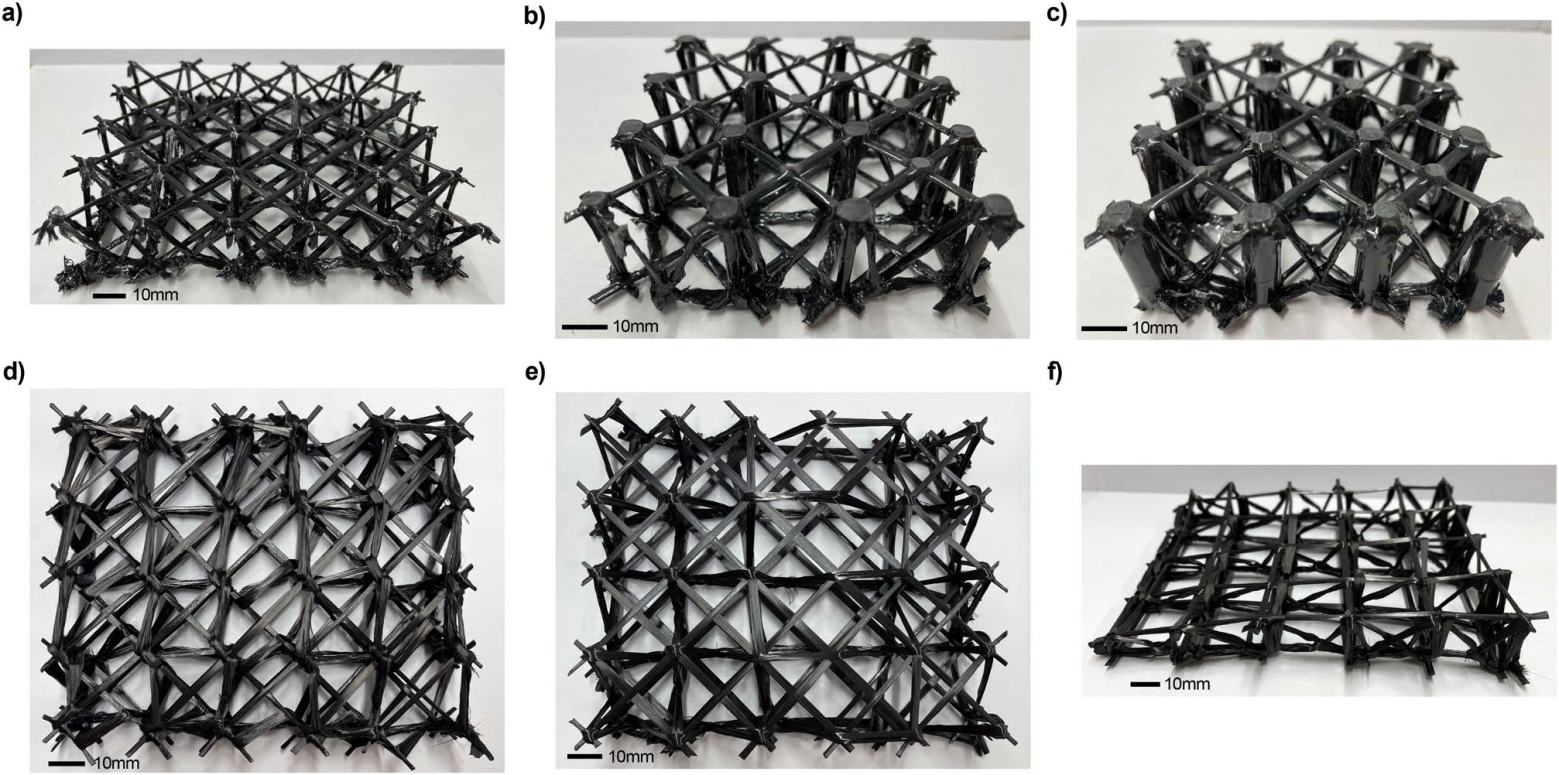
Woven carbon fiber lattices. (**a**) Lattice woven without supporting carbon fiber tubes. (**b**) Lattice woven with supporting carbon fiber tubes with a 3 mm outer diameter. (**c**) Lattice woven with supporting carbon fiber tubes with an 8 mm outer diameter. (**d**) Lattice with an alternative weave pattern. (**e**) Lattice with a differing density weave pattern. (**f**) Lattice woven with a height gradation.

**Figure 2:**
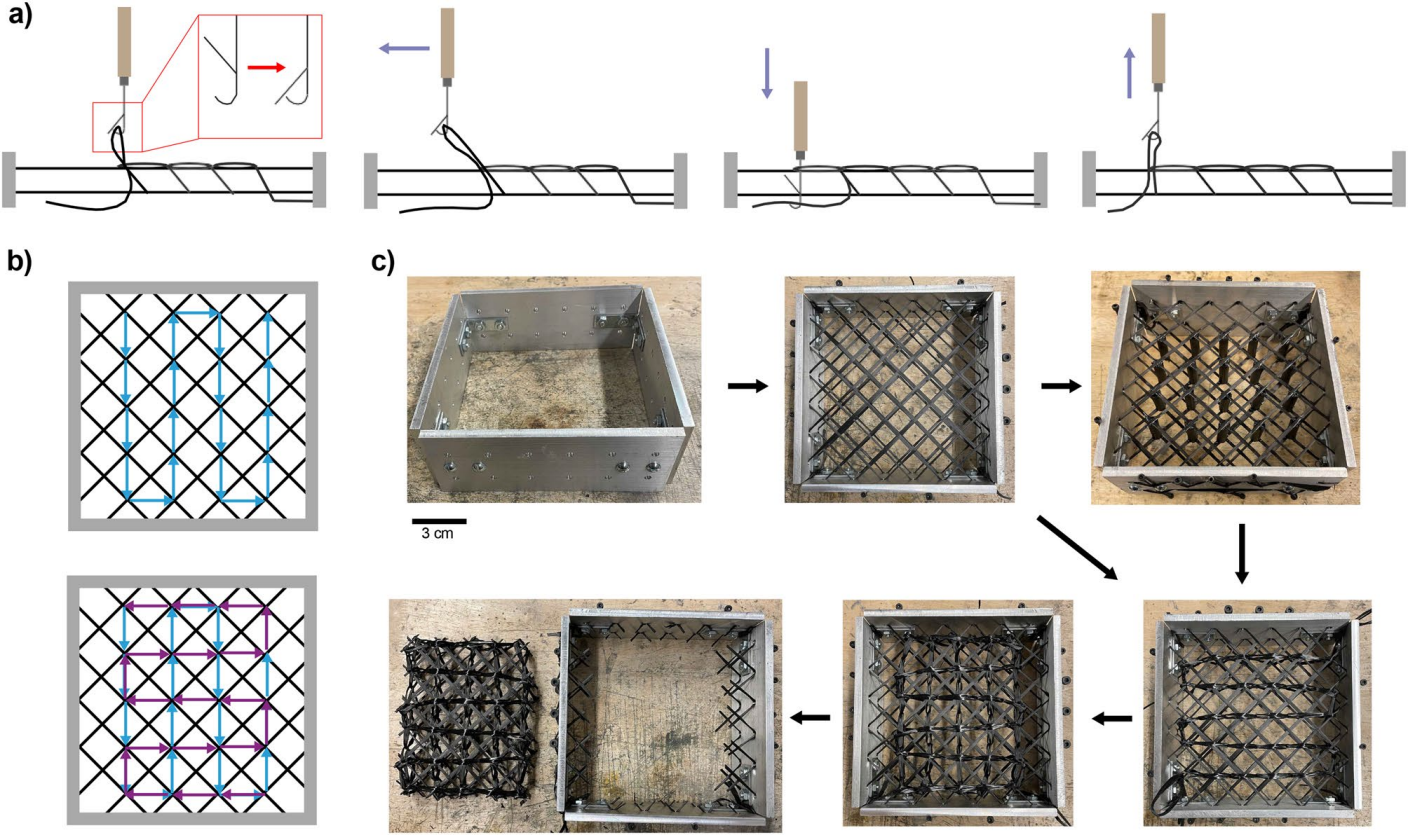
Lattice fabrication. (**a**) Side cross-section schematic of weaving process. (**b**) Top view schematic of weaving process. The blue and purple arrows denote the direction of the weaving. (**c**) Images of the weaving fabrication process.

Three different lattice designs were tested in this study. These include two lattices made with different diameter tubes and a lattice woven without tubes. Examples of these structures, as well as other lattice designs, are shown in Fig. 1. The variety of fabricated structures demonstrates the versatility of the design approach. To test the strength of the structure, the lattices undergo compression testing and the compressive strength is calculated and compared to similar structures found in the literature. These findings are then corroborated by a finite element analysis of each of the three structures (Fig. 2).

### Compression testing

**Figure 3:**
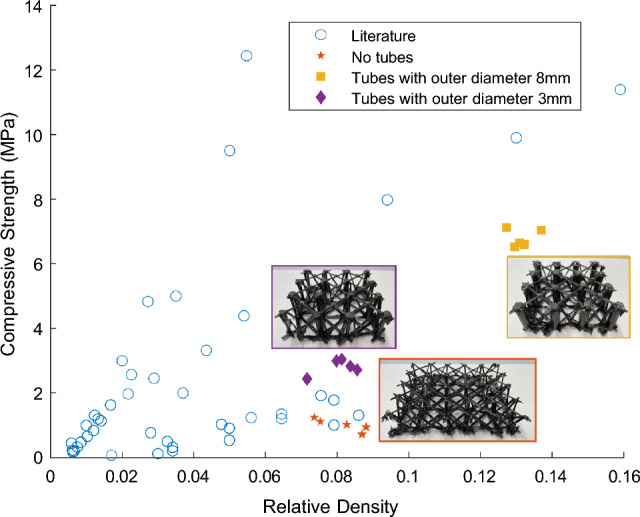
Compression strength comparison. Comparison of the compressive strengths as a function of relative density for similar lattice structures. Specific values for compression strength and relative density are given in Table 1.

**Figure 4:**
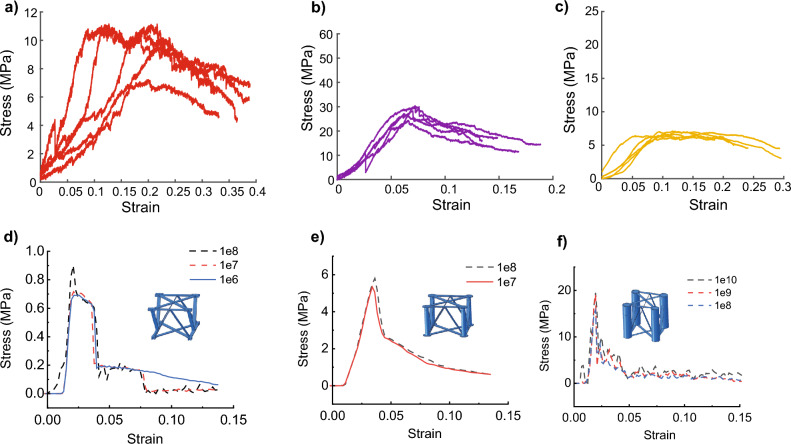
Compression test results. A total of five samples were tested for each lattice geometry. FE model results are shown in blue. (**a**) Stress–strain curves for lattices made without tubes. (**b**) Stress–strain curves for lattices made with tubes with an outer diameter of 3 mm. (**c**) Stress–strain curves for lattices made with tubes with an outer diameter of 8 mm. (**d**), (**e**), and (**f**) The curves of Model 1, 2, and 3, respectively, with different mass scaling. Solid line pattern indicates meeting the criterion of quasi-static condition, which can be verified by comparing the kinetic energy to the internal energy in Abaqus. As expected, decreasing the mass scaling factor can flatten the curves by reducing the pseudo inertial force but increase the run time by a factor of 1/^*√*^*MassScaling* (Table 2). The difference of the curves between experiments and FEM is also due to the their discrepancy in geometry and initial defects.

To characterize the strength of the lattice, compression tests were performed on three different designs to determine their respective compressive strengths. Samples were placed in a universal testing machine (Instron) with a measuring range of 6kN to 600kN and compressed at a rate of 5 mm/min to maintain a quasi-static condition. This gave a maximum force, which was then converted to compressive strength by dividing by the structural cut cross sectional area of the lattice. Relative density was computed by normalizing the density of the lattices by the density of the constituent carbon fiber, coated in epoxy after the initial curing process. The value for the density of the constituent carbon fiber was computed with experimentally found values, and was found to be 1100 kg/m^3^. The results of the compression tests are shown in Fig. 3, where the data is also plotted with reported values from similar lattice structures found in the literature. Stress-strain curves were also computed for each sample type. The stress was calculated by dividing the experimentally found force by the cross-sectional area. These results are shown in Fig. 4. It should be noted that the heights differ between the lattices without tubes and with tubes. This difference occurs during the fabrication process, wherein the two parallel diagonal grids are pulled towards each other during the weaving process for the lattice without tubes. In addition to adding strength, the tubes also keep the vertical distance between the two horizontal grids uniform.

**Table 1:** List of comparable carbon fiber lattices.

Source	Method	Density (kg/m^3^)	Relative Density	Compressive strength (MPa)	Faceplate?
Liu, et al.^21^	3D Printing	29.56	0.056	1.24	Yes
Xu, et al.^17^	Mechanical fasteners	28.78	0.03	0.1198	No
Schneider, et al.^12^	Hot press molding and cut	60	0.037	2	Yes
Yin, et al.^29^	Hot press molding	0.16	0.00585	0.447	Yes
		2.28	0.0645	1.35	Yes
		2.28	0.0645	1.21	Yes
		0.43	0.0123	1.31	Yes
		0.27	0.0103	0.66	Yes
Wang, et al.^15^	Hot press molding	12.78	0.012	0.84	Yes
Dong, et al.^13^	Snap fit	24.48	0.017	0.073	No
		77.76	0.054	4.39	No
		135.36	0.094	7.98	No
		187.2	0.13	9.9	No
		228.96	0.159	11.39	No
Wu, et al.^11^	Hot press molding and cut	79.70	0.0547	12.44	Yes
Eichenhofer, et al.^22^	Pultrusion/extrusion	9.22	0.0066	0.2	Yes
		8.67	0.0062	0.18	Yes
		8.63	0.0061	0.23	Yes
Xiong, et al.^30^	Hot press molding and cut	52.86	0.0341	0.32	No
		77.35	0.0499	0.91	No
		122.61	0.0791	1.78	No
		52.86	0.0341	0.21	No
		77.35	0.0499	0.54	No
		122.61	0.0791	1.01	No
		50.53	0.0326	0.5	No
		73.94	0.0477	1.03	No
		117.03	0.0755	1.92	No
Sun, et al.^10^	Hot press molding	42.16	0.0272	4.83	Yes
Che, et al.^26^	Stitching	7.17	0.0075	0.33	Yes
		22.27	0.0141	1.14	Yes
		35.71	0.0226	2.57	Yes
Fan, et al.^27^	Weaving		0.028	0.77	Yes
	Snap fit		0.086	1.31	Yes
Liu, et al.^16^	Mechanical fasteners		0.0085	0.48	Yes
			0.0133	1.2	Yes
			0.0168	1.63	Yes
			0.0217	1.97	Yes
			0.0289	2.46	Yes
			0.0435	3.32	Yes
Finnegan, et al.^5^	Snap fit		0.01	1	Yes
			0.02	3	Yes
			0.035	5	Yes
			0.05	9.5	Yes
This work	Weaving	89.68 ± 7*.*280	0.0813 ± 0*.*0066	1.01 ± 0*.*197	No
		88.66 ± 5*.*947	0.0804 ± 0*.*0054	2.80 ± 0*.*243	No
		144.86 ± 3*.*980	0.1314 ± 0*.*0036	6.79 ± 0*.*272	No

**Table 2:** Comparison among simulations and corresponding experiments from Fig. 4 in terms of peak values, wallclock run time, and quasi-static status. The simulation run time is proportional to 1/*√MassScaling*. The FE Model 3 failed to reach a quasi-static loading due to an unknown bug. The geometric difference, defects difference, and mass scaling are believed to be the major reason of the in-consistence between FEM and experiments.

	Model 1			Model 2		Model 3		
	d) le8	d) le7	d) le6	e) le8	e) le7	f) le10	f) le9	f) le8
Peak value (Mpa)	0.9005	0.7161	0.6902	5.8011	5.3372	19.4724	19.1642	15.7251
Wallclock time (s)	~ 553	1947	6407	3845	12290	454	1388	4290
Quasi-static	N	N	N	N	Y	N	N	N

**Figure 5:**
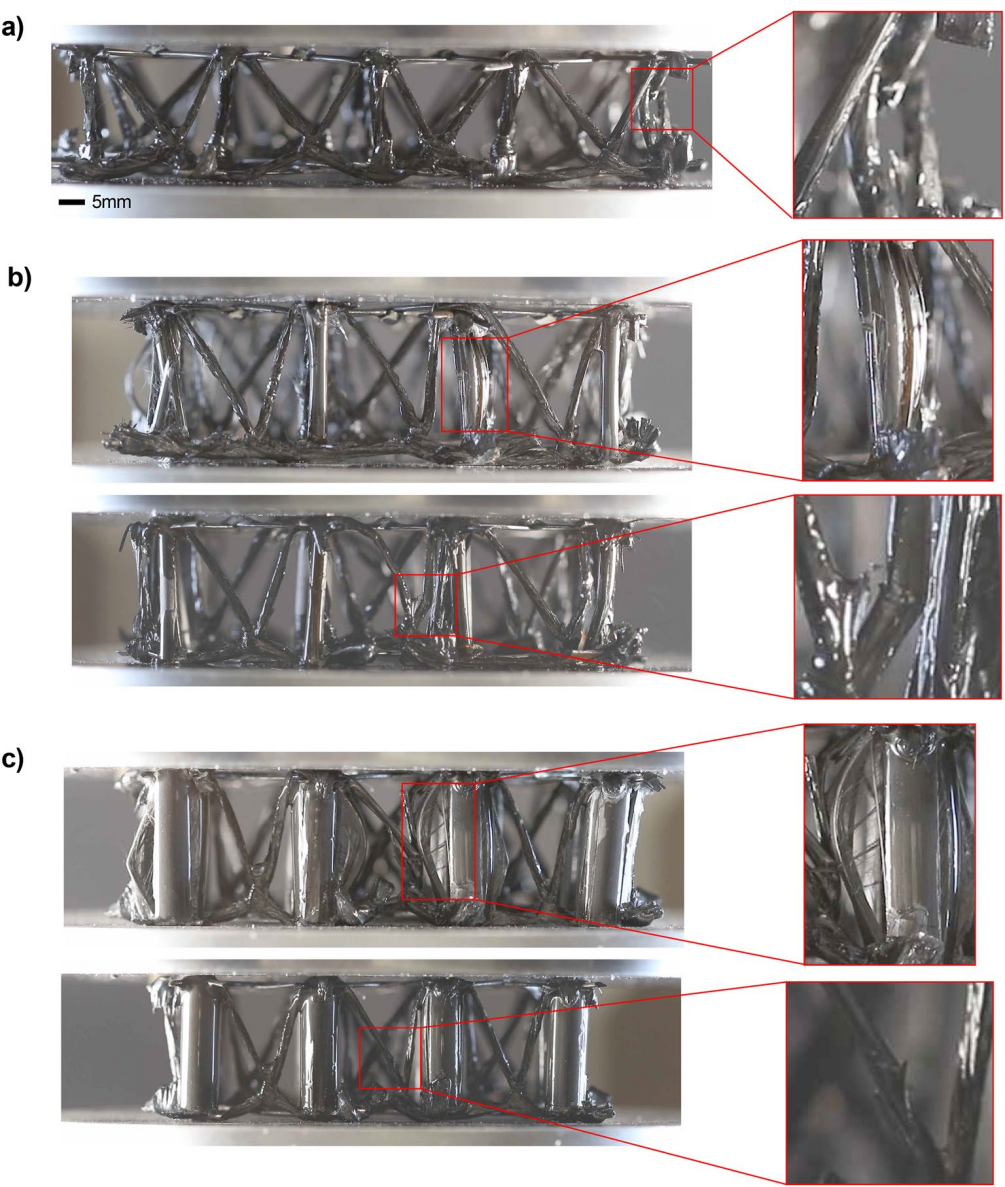
Failure modes. (**a**) Failure mode of a lattice made without tubes. The inset shows that failure occurs at the diagonal truss. (**b**) Failure mode of a lattice made with tubes with an outer diameter of 3 mm. The inset shows that failure occurs at both the tube and the diagonal truss. (**c**) Failure mode of lattice made with tubes with an outer diameter of 8 mm. Failure occurs at both the tube and the diagonal truss.

Compared to similar lattices, the lattices with the added carbon fiber tubes show a comparable performance to similar designs (a complete listing of the data points shown in Fig. 3). For example, for the lattice made without tubes, the data points are similar in range to four other lattices found in the literature. The lattice made with tubes of outer diameter 3mm is about the same relative density as these four points, but has a higher compressive strength. The lattice made with tubes of outer diameter 8mm is not close in range to the other lattices, but has a higher compressive strength than all but 5 of the lattices found in the literature. One detail to note is that many other similar designs utilize a faceplate when testing their lattices. This adds a significant amount of structural support. The lattice presented here was specifically designed without a faceplate so as to provide maximum flexibility with the weaving design. During compression tests of the lattice designs, videos were captured to examine the main failure modes of the structures (movies S1, S2, S3). For the lattice made without tubes, the main failure mode was the buckling of the diagonal struts, but there was also some delamination of the struts from the horizontal, diagonally arranged grids, which caused the internal structure to slide. As shown in Fig. 5, there are two failure modes for the woven lattice with the vertical tubes; buckling of the carbon fiber tubes and buckling of the diagonal struts. In compression, the tubes are the main supporting feature, but in bending or in out-of-plane compression, a different failure mode is expected and we believe the supporting woven structure should provide the needed support.

### Finite element analysis (FEA)

Corresponding FE simulations were performed with the commercial finite element package ABAQUS/Explicit (2020, Dassault Systèmes Simulia Corp., USA) to obtain insights into the compressive behaviors of the carbon fiber/epoxylattice structures. To simulate the failure and fracture of the lattices, Hashin’s damage theory, a well-accepted model for fiber-reinforced composite materials^16^, is used. This theory considers the criteria of the four failure modes respectively:

$$ F_{1}^{T} = \left( {\frac{{\sigma_{11} }}{{S_{1,T} }}} \right)^{2} + \left( {\frac{{\sigma_{12} }}{{S_{11} }}} \right)^{2} \le 1.0,\;{\text{Fiber}}\;{\text{tension}}\;\left( {\sigma_{11} \ge 0} \right) $$

$$ F_{1}^{C} = \left( {\frac{{\sigma_{11} }}{{S_{1,C} }}} \right)^{2} \le 1.0,\;{\text{Fiber}}\;{\text{compression}}\;\left( {\sigma_{11} < 0} \right) $$

$$ F_{2}^{T} = \left( {\frac{{\sigma_{22} }}{{S_{2,T} }}} \right)^{2} + \left( {\frac{{\sigma_{12} }}{{S_{12} }}} \right)^{2} \le 1.0,\;{\text{Matrix}}\;{\text{tension}}\;\left( {\sigma_{22} \ge 0} \right) $$

$$ F_{2}^{C} = \left( {\frac{{\sigma_{22} }}{{2S_{23} }}} \right)^{2} + \left[ {\left( {\frac{{S_{2,C} }}{{2S_{23} }}} \right)^{2} - 1} \right]\;\frac{{\sigma_{22} }}{{S_{2,c} }} + \left( {\frac{{\sigma_{12} }}{{S_{12} }}} \right)^{2} \le 1.0,\;{\text{Matrix}}\;{\text{compression}}\;\left( {\sigma_{22} < 0} \right) $$

where *σ*_11_, *σ*_22_, and *σ*_12_ denote the stresses. *S*_1*,T*_ , *S*_1*,C*_, *S*_2*,T*_ , and *S*_2*,C*_ are tensile/compressive strengths in corre-sponding directions. *S*_12_ and *S*_23_ are shear strengths. The values of these variables are given in Table 3.

**Table 3:** Mechanical property of the carbon fiber/epoxy composite material. Variable *ρ* is mass density, *E*_*i*_, *ν*_*i j*_, *G*_*i j*_, *S*_*i,T/C*_,and *S*_*i j*_ are Young’s moduli, Poisson’s ratios, shear moduli, normal strengths, and shear strengths in corresponding directions. *U*_*i,T/C*_ are tensile/compressive fracture energy in the corresponding directions^32^.

Ρ (g/cm^3^)	*E*_1_ (MPa)	*E*_2_ (MPa)	*v*_12_	*v*_23_	*G*_12_ (MPa)	*G*_23_ (MPa)	*S*_1,*T*_ (MPa)	*S*_1,*C*_ (MPa)	*S*_2,*T*_ (MPa)	*S*_2,*C*_ (MPa)	*S*_12_ (MPa)	*S*_23_ (MPa)	*U*_*i,T/C*_ (N/mm)
1.22	140000	9000	0.28	0.49	5400	5400	2570	1570	63.4	266	95.8	95.8	12.5

**Figure 6:**
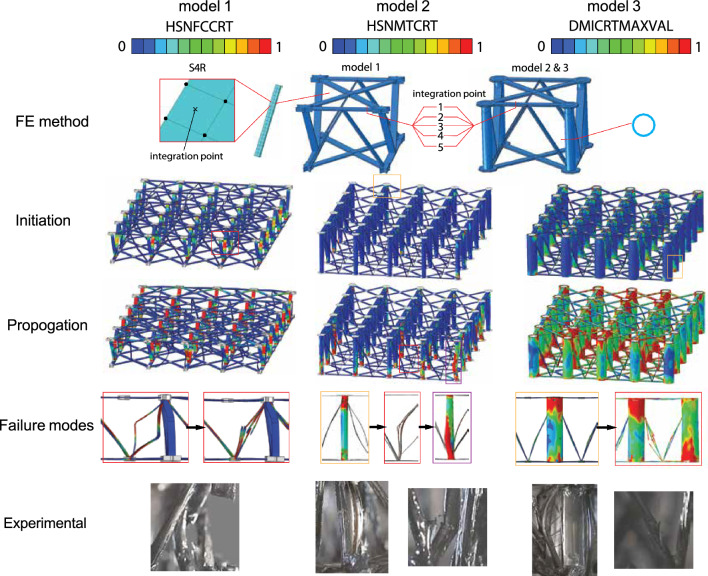
Finite-element (FE) modeling of the carbon fiber/epoxy lattice structures. Hashin damage variables HSNFCCRT (fiber direction compression criterion value), HSNMTCRT (transverse direction tensile damage criterium value), and DMICRTMAXVAL (maximum damage initiation value) are contoured respectively in models 1–3 due to their different failure modes. Red squares denote compressive buckling and cracking in the diagonal struts, yellow squares mean tube transverse tensile failure, and purple squares indicate tube buckling. The bottom row shows the corresponding failure observed in the experimental samples.

Three different FE lattices are built to match the geometries of the fabricated samples in Fig. 5. In each of these models, a total of 4 x 5 repeating units were used, and the cured carbon fiber strand is approximated to be a uniform beam with a sectional size of 2.2 mm by 0.5 mm (Fig. 6). Overlapped areas (knots) of different carbon fiber ribbons in the lattices are assumed to be non-destructible since they are much stiffer and are simulated with the isotropic carbon fiber/epoxy model^16^. ABAQUS/Explicit was found to be a better tool than ABAQUS/Standard to simulate complicated nonlinear and failure problems in terms of convergence and computational cost^31^. To achieve a quasi-static condition, all compressions are carried out in at least 60 seconds with a 5 mm/min loading rate. The total number of S4R elements used in these three models are 13032, 42088, and 35240, respectively, with 5 integration points in the thickness direction (Fig. 6) for each element. To accelerate the simulation, a mass scaling factor of 1e10 1e7 is applied. Mass scalings speed up the simulation by increasing the time span of each step but will result in overestimated inertial forces that lead to jagged plots, especially when the lattice begins to crack.

However, even though the FEM stress-strain curves cannot describe the mechanical properties of carbon/fiber lattices well, the simulation reveals the failure modes clearer than the experiments considering the filming conditions. In the FEM model without supporting tubes (Model 1 in Fig. 6), the compressive loading leads to Euler buckling of the struts and then cracking and lattice failure (highlighted with red squares in Fig. 6, movie S1), while Model 2 and 3 experience transverse or matrix tensile failure at the top and bottom of the tubes before the struts buckling. In movie S2 and movie S3, however, corresponding failure modes happen almost during the same period. For the tubes with a higher slenderness ratio in model 2, compressive column buckling takes place in the tubes after a certain level of material degradation, while tubes in model 3 do not buckle due to a larger radius and larger wall thickness. This difference can also be observed in videos S1, S2, and S3.

## Discussion

Here, a novel carbon fiber lattice was developed using crocheting and sewing techniques. Using a jig with two parallel carbon fiber grids as a scaffold, prepreg carbon fiber tow was woven through the grid in a rectangular pattern using a latch hook. Additional structures with vertical carbon fiber tubes were also studied. This process yields a cellular lattice with a relatively high compressive strength to density ratio and demonstrates a comparable performance to other similar lattice structures found in the literature. For example, the lattice made without tubes performs simlarly to four lattices in the literature. For the same four values, the lattice with the tubes of outer diameter 3mm has the same relative density, but has a higher compressive strength by about 1MPa. The lattice with tubes of outer diameter 8mm is not close in value to any structures found in the literature, but has a higher compressive strength than most of the cited sources. Additionally, a FE analysis is performed as well to validate the experimental results. Though the peaks occur much higher and at a lower strain, we believe that the difference can be attributed to error introduced in the manual fabrication process.

However, while other approaches require the presence of a face plate, the absence of a face plate here allows for a more free-form fabrication approach. Additionally, the weaving process in this approach allows for the possibility of automation. While the weaving could be easily automated, the additional steps of adding in vertical tubes and removing excess carbon fiber from the edges will be more difficult to automate. Some other limitations of this method include the variability in the struts. For example, the diameter of the struts are not uniform and the amount of added epoxy can differ from strut to strut. Additionally, this method only allows for lattices of one unit cell in height to be made due to the weaving process. Future study of this fabrication should focus on altering the fabrication process so as to be robotically manufactured. Other future studies should also focus on alternate lattice designs. These can include designs where the struts consist of twisted pieces of tow, or with multiple pieces of tow. Different weaving patterns could also be explored, in order to find the most efficient design. With the flexibility of this design process, numerous future iterations can be explored.

### Supplementary Information

Supplementary Video S1.

Supplementary Video S2.

Supplementary Video S3.

Supplementary Information 1.

## Data Availability

The datasets used and/or analysed during the current study available from the corresponding author on reasonable request.
